# Challenges in defining thresholds for health effects: some considerations for asbestos and silica

**DOI:** 10.3389/fepid.2025.1557023

**Published:** 2025-03-17

**Authors:** Julie E. Goodman, Lorenz R. Rhomberg, Samuel M. Cohen, Kenneth A. Mundt, Bruce Case, Igor Burstyn, Michael J. Becich, Graham Gibbs

**Affiliations:** ^1^ Gradient, Boston, MA, United States; ^2^ Havlik-Wall Professor of Oncology, Department of Pathology, Microbiology, and Immunology, and the Buffett Cancer Center, University of Nebraska Medical Center, Omaha, NE, United States; ^3^ University of Massachusetts, Amherst, MA, United States; ^4^ McGill University, Montreal, QC, Canada; ^5^ Drexel University, Philadelphia, PA, United States; ^6^ University of Pittsburgh School of Medicine, Pittsburgh, PA, United States; ^7^ Private Consultant in Epidemiology and Occupational Health, Eastbourne, United Kingdom

**Keywords:** thresholds, asbestos, elongate mineral particles, silica, mesothelioma, silicosis

## Abstract

This paper summarizes several presentations in the Thresholds in Epidemiology and Risk Assessment session at the Monticello III conference. These presentations described evidence regarding thresholds for particles, including asbestos and silica, and cancer (e.g., mesothelioma) and noncancer (e.g., silicosis) endpoints. In the case of exposure to various types of particles and malignancy, it is clear that even though a linear non-threshold model has often been assumed, experimental and theoretical support for thresholds exist (e.g., through particle clearance, repair mechanisms, and various other aspects of the carcinogenic process). For mesothelioma and exposure to elongate mineral particles (EMPs), there remains controversy concerning the epidemiological demonstration of thresholds. However, using data from the Québec mining cohort studies, it was shown that a “practical” threshold exists for chrysotile exposure and mesothelioma. It was also noted that, in such evaluations, measurement error in diagnosis and exposure assessment needs to be incorporated into risk analyses. Researchers were also encouraged to use biobanks that collect specimens and data on mesothelioma to more precisely define cases of mesothelioma and possible variants for cases of all ages, and trends that may help define background rates and distinguish those mesotheliomas related to EMP exposures from those that are not, as well as other factors that support or define thresholds. New statistical approaches have been developed for identifying and quantifying exposure thresholds, an example of which is described for respirable crystalline silica (RCS) exposure and silicosis risk. Finally, the application of Artificial Intelligence (AI) to considering the multiple factors influencing risk and thresholds may prove useful.

## Introduction

Risk assessors have criticized epidemiologists for failing to account for threshold effects in data analyses, indicating that the incorporation of thresholds is needed to make epidemiology more policy-relevant (1).

With respect to arguments that have been made in favor of linear, no-threshold models of cancer risk, it has been suggested that given the usually incomplete understanding of the underlying biological reasons for the existence of background cases, it is hard to refute the possibility of additivity to this background and hence induction of responses at even the lowest doses. A resulting policy perspective is that—to be precautionary—lack of thresholds should be presumed to ensure safety from effects for which low-dose risks cannot be ﬁrmly refuted (2).

However, the reason that an increasing dose-response pattern is observed for various types of cancers (i.e., higher cancer incidence at higher exposures) has traditionally been thought of as due to one of two basic causes:

 • Stochastic events: In a stochastic event, an event either does or does not happen, the site of action is specific, and an accumulation of “hits” precipitates the toxic response. This term is generally used in reference to genotoxic carcinogens, with the “hits” being somatic mutations and a malignancy is generated when any cell acquires a set of such mutations that cause it to behave as a malignant cell. Because the all-or-none events are possible from single molecules (albeit increasingly unlikely at lower exposures), a threshold would generally not be expected.
 • Tolerance distributions: Individuals at risk vary in their abilities to tolerate stresses or damage, and higher doses exceed the individual thresholds of an increasing fraction of the population, leading to more responses. This term has generally been applied to noncancer toxicity, and a threshold of insufficient collective effect to cause adverse reactions is often assumed.

It is useful to consider that the above distinction hinges on how the agent affects its targets:

 • If *individual molecules* (or fibers) affecting individual cells generate a critical effect, then the stochastic event model should describe the process, with potential target cells either affected or not in an all-or-none way, the probability of which (but not the magnitude) is a function of dose. Whether or not the event happens in cell “A” is independent of what happens in nearby cell “B.” That is, the concentration of the causative agent is only affecting the probability that events happen, but the *events are independent* of one another.
 • If the critical effect instead depends on the *collective impact* of all the units of the agent (molecules or fibers), then the tolerance distribution model applies. That is, the physiological reaction is related to the concentration of the causative agent, which varies continuously and has an effect that varies in magnitude dependent on its level. Each individual molecule (or fiber) of the causative agent is only generating a small fraction of the total response, but it is the sum of these small amounts that has consequences, not each individual molecule's individual actions.

In sum, the distinction is between the probability of all-or-nothing localized events precipitating the adverse change vs. the collective sum of all the small events' consequences exceeding some tolerable level (3–5). Both of these mechanisms have thresholds and suggest that if thresholds are not found in dose-response curves in epidemiology studies, they are likely obscured for various reasons.

## The biologic basis for thresholds in carcinogenesis

There are many examples of thresholds in normal biology and therapeutics. For example, humans need oxygen to live, but exposure to 100% oxygen (O_2_) can cause blindness (retrolental fibroplasia) in premature babies (6) and lung damage in adults (7). In addition, the presence of thresholds can be inferred by the numerous defense mechanisms in cells, such as DNA repair, immune response, metabolism, and others, that protect against adverse responses.

With respect to carcinogenesis, it is known that several genetic alterations are required for cancer formation, DNA replication fidelity is not 100%, cancer arises from a stem cell population, cancers are clonal, and carcinogenesis is stochastic process (8–10). Thus, there are essentially only two ways to increase the risk of carcinogenesis: increase the rate of DNA damage per cell division or increase the number of cell divisions. With respect to the latter, this can be a result of an increase in cell births via direct mitogenesis or toxicity and regeneration, or a decrease in cell deaths by inhibiting apoptosis or cell differentiation.

Cohen (9) and Cohen et al. (8) demonstrated that there are several modes of action for human carcinogens, including immunosuppression, estrogenic activity, DNA reactivity, and increased cell proliferation. The mode of action for asbestos in mesothelioma has not been fully established in toxicology, with various hypotheses proposed [e.g., (11)]. Asbestos is not DNA reactive, immunosuppressive, or estrogenic, so its mode of action will be cytotoxicity with regenerative proliferation. Consistent with this is the recent proposal by Carbone et al. (12), who argued that asbestos/fiber carcinogenesis occurs because of the chronic inflammatory process that is induced in mesothelial cells, accompanied by the secretion of HMGB-1 proteins that “activates autophagy…that helps mesothelial cells survive asbestos exposure.” If the Carbone hypothesis is correct, there are several threshold-based stages in mesothelial carcinogenesis. In particular, the inflammation itself requires an exposure threshold to become chronic (13). Also, a threshold in mesothelioma can be observed from the balance between the intensity of inflammation and the intensity of the cell survival proteins, which are secreted because of the exposure (and potential mechanical damage that rigid asbestos fibers can produce in the cells).

Even without accepting the HMGB-1 hypothesis, we can argue that, if the mode of action of asbestos is cytotoxicity with regenerative cell proliferation, this process would also require exposure above a threshold dose for the development of disease. Thus, no excess in cancer incidence is expected if the dose is below a threshold. A prototypic example is chloroform (14), which causes liver and kidney cancers in rodents via cytotoxicity/regeneration at very high exposures. High doses of chloroform are also toxic to the human liver and kidney, but only at the high doses used in anesthesia; there is no evidence that chloroform causes cytotoxicity at the much lower exposures occurring through drinking water. In the same vein, asbestos requires a threshold. Unlike chloroform, however, one must account for accumulation of asbestos over time. A typical reaction to particulates in the lung at high exposures will lead to cytotoxicity, inflammation, and reparative regeneration, with a threshold dose-response.

## Issues with mesothelioma diagnoses

Diagnostic error for mesothelioma can contribute to misclassification of risk in either direction, and much of the historical cohort and case-control study data that informs on risk did not use pathology validation, relying instead on death certificate data and hospital records. Where pathology was available, it relied histologically mainly on Hematoxylin and Eosin (H & E) staining. More recent studies were improved by the advent, in the late 1990s, of immunohistochemical (IHC) markers of mesothelial cell origin, although these vary in sensitivity and specificity, as do markers of differential diagnoses including various carcinomas metastatic to the pleura. This gradual evolution and improvement in certainty of pathology diagnosis attributable to IHC advances is described elsewhere (15). Current standard international pathology practice for both morphology (using H & E) and IHC is maintained by the International Mesothelioma Interest Group (16).

## Statistical issues in identification and estimation of thresholds from epidemiological data

Occupational epidemiology studies clearly demonstrate increased risk of silicosis among workers exposed to respirable crystalline silica (RCS); however, few have quantiﬁed with any precision the exposure thresholds at which risk signiﬁcantly increases. For example, evidence from the German Porcelain Workers Study, in which silicosis cases were defined as those with B-reader International Labour Organization (ILO) scores ≥1/1, suggested thresholds for both cumulative and average exposures based on simple Cox proportional hazards analysis by exposure categories (17). However, there were only 40 silicosis cases (18) and analysis by categories is fraught with challenges, including introduction of differential exposure misclassification (19, 20).

Using these data, Morfeld et al. (21) applied a likelihood proﬁle estimation procedure for Cox regression analyses and estimated the best-ﬁt average exposure intensity threshold but detected no threshold using estimated cumulative exposure; confidence intervals (CIs) were obtained using a bootstrap. This work was extended by application of segmented Cox regression that provided a maximum likelihood estimate and CI for the best-fitting threshold. In the new analysis, silicosis cases were deﬁned as having ILO scores ≥1/0 and were each matched to four controls. RCS exposure was based on estimated annual average intensity for each worker over their ﬁrst two and ﬁve years of employment, as comparing the effects of continuous and discrete exposures tended to be highest in the ﬁrst several years of employment, as well as cumulative exposure over their entire employment (22). While applying such a threshold-seeking analytical approach returns the exposure value (and 95% CI) at which risk of the event statistically significantly departs from no excess risk [i.e., hazard ratio (HR) = 1.0], as well as coefficients (i.e., slopes) for modeled segments before and after the threshold estimate, it does not inform the shape of the dose-response function between the reference (typically the lowest exposure) group and the estimated threshold. It is unlikely a straight line; however, it also may be inestimable, as very few cases typically are observed below the threshold, as would be expected if the threshold estimate is accurate. The cases with very low associated exposure likely reflect exposure misclassification (22).

When segmented regression (any regression model) is used to identify a threshold (or complex shapes), a qualitatively and quantitatively incorrect exposure-response shape and threshold may be reported if measurement error is ignored. This was illustrated in simulated data that mimics a Québec cohort of miners and millers, and the relationship that may exist within that population between exposure to dust and fibers. It was argued that it is impossible to draw conclusions about the shape of the exposure-response and a threshold without quantitatively adjusting for measurement error in exposure and allowing for such thresholds to exist within the statistical model. Developing accessible statistical methods that do this would constitute an important advance on current practices in epidemiology.

## A practical example

In their seminal paper estimating lung cancer and mesothelioma asbestos risks, Hodgson and Darnton (23) asserted, “Direct statistical conﬁrmation of a threshold from human data is virtually impossible.” This is inarguably true but does not mean that there *is* no threshold.

Direct observation of human exposure and disease within an epidemiology study could provide evidence of a generally “safe level of exposure”—a practical rather than a statistical concern. A review of exposure and disease in Québec chrysotile miners, millers, and factory workers; their families; and their neighbors can be used as an example of the kinds of analyses—and problems—that are possible.

The Québec chrysotile mining area can be subdivided into three areas. The first area, known in the literature as “Asbestos,” is near the town of that name, which was recently renamed “Val des Sources.” It is better referred to as the “Jeffrey Mine,” which is the only mine in the area and the world's second largest chrysotile mine. Also located in the town of Asbestos was a factory, operated by Johns-Manville, that used crocidolite asbestos in some of the products manufactured there.

The second and third areas, often referred to collectively as the “Thetford Mines,” are two sets of a number of mines each, located between 60 and 100 km northeast of the Jeffrey Mine. Although these are usually referred to as a single group, they are best looked at separately as

A. The originally exploited mines, such as “Bell”—the “original complex.” This “localized area of five mines (Area A)” have highest tremolite content (24, Chatfield et al., submitted), and
B. The third area, usually referred to as within “Thetford Mines,” consists of about 15 mines farther away from that town and its original complex (Area A) at Thetford Mines. This third area has been called “Area B” (24) or the “peripheral complex” (25).

The team led by the McDonalds at McGill University conducted studies of Québec chrysotile miners, millers, and associated factory workers starting in 1966 and were followed to the end of 1992 (25, 26). Thirty-three mesothelioma cases were identified among male miners and millers exposed principally to chrysotile and (possibly non-asbestiform) tremolite EMPs. A case-control analysis within the Thetford Mines region reported a more than two-fold excess for men who had worked at least 20 years in the original complex of mines and mills with higher levels of tremolite (25). At the Johns-Manville factory in Asbestos, where all 708 employees were potentially exposed to crocidolite and/or amosite, there were 553 deaths with five mesotheliomas—3.5 times higher than among the primarily chrysotile-exposed miners and millers at the Jeffrey Mine, despite cumulative exposure an order of magnitude lower.

Study of mesothelioma in miners, millers, and factory workers was complicated by the fact that the mesothelioma diagnoses in five cases was of low confidence and only of moderate confidence in 14. In addition, six cases had worked there for five years or less and had possible alternative asbestos exposures in other work.

After removing the low-confidence diagnoses and short-duration cases from the analysis, 23 cases with all data available remained for exploratory analyses for a practical threshold. These analyses were run by Andrey Korchevskiy using data from Dagbert (27) that were provided by Bruce Case; the original midget impinger dust measurements had been provided by Graham Gibbs to Dagbert (27). Dr. Korchevskiy's preliminary results, using those dust measurements, which were converted to fibers/cc-years (f/cc-years) based on paired membrane filter samples, with raw pair data obtained from Dagbert (27), showed an average cumulative exposure in the cohort of approximately 500 f/cc-years, closely approximating L. Darnton's estimated average exposure of 600 f/cc-years (28). The 23 mesothelioma cases had a greater mean exposure of over 1,000 f/cc-years, and the lowest of the 23 had an estimated exposure of 135.7 f/cc-years. Using chrysotile lung content where available (17 of the 23 cases), a “central tendency” of cumulative exposure to chrysotile at 148.8 f/cc-years was reported, but a CI is difficult to establish. It is important to understand that these modelled exposure estimates apply to a selective subsample of cases, and are exploratory, but they do point to a “threshold” value of 100 f/cc-years or greater, close to what others have estimated (29, 30).

Case et al. (31) examined ten women with mesothelioma identified in the Québec mining areas among all female cases (aged >50 years) diagnosed in Québec hospitals during 1970–1989. No cases were identified around Asbestos (Jeffrey Mine); all ten were near Thetford Mines. These were matched with 150 area controls. Five of the ten cases were found to be asbestos workers and nine were living with asbestos workers. A complex exposure reconstruction based on five data sources, as outlined by Camus et al. (32), was used to estimate exposures for the ten women, with a result averaging 226.1 f/cc-years (range: 84.5–525.6 f/cc-years). Although the exposure estimation was completely different, the results appear comparable with those for the chrysotile miners and millers. Two women had lung fiber content analyzed; both had crocidolite and amosite in their lungs from occupational exposures in a small bag repair shop.

To summarize, based on the available data for mesothelioma cases in the chrysotile mining areas of Québec, there was minimal risk at well above the equivalent of 100 fibers total EMP/cc. Sources of uncertainty, however, are many, including the mesothelioma diagnosis itself, as well as the known issues around exposure assessment. Further analysis will be necessary to determine the degree of certainty with which comparisons between chrysotile mining and milling cohort exposure values can be compared with other cohorts. However, the relatively small numbers of mesothelioma deaths, with fewer expected over time, severely limits statistical modelling approaches.

## Other theoretical and empirical models

In addition to studies with the Québec cohort, there are several other theoretical and empirical models of EMP exposures and mesothelioma risk. These are described briefly below and in more depth by Goodman et al.^1^

A theoretical model was proposed for the development of mesothelioma in humans, assuming that several counteracting factors are involved in the process, as in Carbone et al. (12). In this model, the probability of mesothelioma depends on the probability of inflammation, cell death because of cytotoxicity, and induction of a process that promotes cell survival. Graphing this model results in a hockey-stick-shaped curve, which has a clear threshold.

Several models were tested on the epidemiological data for mesothelioma in chrysotile cohorts. The original data analyzed by Darnton (28, 33) was expanded to include new data from the International Agency for Research on Cancer for the Russian miners and millers cohort. Statistical variability for the reported datapoints was introduced to account for uncertainty in exposure measurements and mesothelioma observations. The Monte Carlo simulation was applied to the data to check if a linear threshold model would fit the epidemiological information better than a linear non-threshold model. The simulation study demonstrated that 72% of fitted models confirmed the presence of thresholds for chrysotile cohorts. The average threshold value of 25.6 f/cc-years was found (95% CI: 24.2–27.1), with the 5th and 95th percentiles being 3.3 and 52.9 f/cc-years, respectively.^1^

Also, the model previously developed by Korchevskiy and Korchevskiy (34) was tested on the chrysotile epidemiological data. It was demonstrated that a threshold-based model can be combined with the Peto equations for the relationship between mesothelioma mortality and age. The exposure intensity threshold of about 2 f/cc was suggested, with a threshold cumulative exposure of up to 90 f/cc-years.

We also note that Schaeffer et al. (35) developed a filter model based on the Lagrangian Poisson Process for chromosome aberrations from radiation exposure and applied it to other carcinogens. Using this model with data for non-textile chrysotile cohorts, the threshold model was again supported, with a threshold level of 162 f/cc-years. Further studies are needed to determine the threshold values for other mineral types of fibers, but an established relationship between potency factors would suggest a threshold for Libby amphiboles of 4.3 f/cc-years, for amosite of 1.04 f/cc-years, and for crocidolite of 0.25 f/cc-years.^1^

A threshold is also demonstrated with empirical models based on the Surveillance, Epidemiology, and End Results (SEER) Program cancer data and asbestos consumption data. SEER is a US-based cancer registry supported by the National Cancer Institute (https://seer.cancer.gov/), and asbestos consumption data are available from a US Geological Survey annual report of domestic production and use of asbestos (36). Moolgavkar et al. (37) originally modelled asbestos consumption and mesothelioma risk using a two-stage clonal expansion model. This model was modified and demonstrated a good fit with the inclusion of a threshold.

Finally, with respect to environmental exposures, there are a number of more qualitative studies of environmental exposures to asbestos in the neighborhoods of asbestos mines and asbestos cement plants that used crocidolite with chrysotile in pipe manufacture. These include the area around the Kubota asbestos cement plant in Japan (38), where mesothelioma risk appears to be related to the distance from the plant as point source. Similar findings were determined around the Casale Monferrato plant and others in Italy (39, 40), and near the Johns-Manville pipe plant and other asbestos industries in Jefferson Parish, Louisiana (41). A classic example is work showing mesothelioma cases arising after brief but possibly intense exposures to crocidolite among those living near the mine in Wittenoom, Western Australia (42, 43). Unfortunately, none of these studies provide confident measures of actual exposure levels, none especially inform as to the presence or absence of a threshold generally, and all apply principally to crocidolite. It is also a mistake to generalize “environmental” exposures as being “low-dose” exposures, as often they are not (44). As such, results of these studies are not necessarily inconsistent with threshold models.

## A path to asbestos threshold determination for mesothelioma using biobanks

Determining thresholds of asbestos exposure that can lead to mesothelioma is a complex and critical task, as it involves the assessment of various factors, including asbestos types, ﬁber size, duration of exposure, genetic susceptibility, and individual health conditions. Traditional threshold-based exposure limits, such as permissible exposure limits (PELs), are in place for occupational safety, but they do not necessarily directly correlate with health risks, such as mesothelioma. A multi-faceted approach to help determine more accurate thresholds for asbestos exposure leading to mesothelioma can be accomplished using the National Mesothelioma Virtual Bank (NMVB) (https://mesotissue.org/) as a foundation. Avenues of research and potential tools include:

 • Longitudinal cohort studies: Researchers can conduct long-term cohort studies involving individuals with documented asbestos exposures. These studies can monitor the health of exposed individuals over an extended period, collecting data on the type and duration of exposure, as well as genetic and health factors. These studies can also provide insights into cumulative dose-response relationships and help identify critical exposure thresholds.
  ○ *Example Innovation:* Visonà et al. (45) conducted a study of an Italian cohort with lung ﬁber digests by scanning electron microscopy/energy-dispersive x-ray spectrometry (SEM-EDS). The authors reported that their results indicate an asbestos threshold associated with lung fibrosis, pleural plaques, and ferruginous bodies in patients with mesothelioma.
 • Advanced biomarkers and genetic proﬁling: Utilizing advanced biomarker analysis and genetic proﬁling, researchers can identify individuals with a higher susceptibility to mesothelioma due to genetic factors. Understanding asbestos exposure thresholds in mesothelioma should be facilitated by DNA/RNA, protein, epigenetic, and proteogenomic biomarkers. This information can help reﬁne exposure thresholds for individuals with speciﬁc genetic markers that increase their risk.
  ○ *Example Innovations:* ToxicoGenomica (https://www.toxicogenomica.com/) and The Cancer Genome Atlas Project provided public access to genomic data on 74 cases of pleural mesothelioma and were supplied by hospitals and medical centers participating in the NMVB. This analysis was published by Hmeljak et al. (46). In addition, miRNA studies have shown promise in identifying cellular and diagnostic changes related to asbestos in mesothelioma cell lines and resected surgical tissues (47, 48).
 • Artiﬁcial intelligence (AI) and machine learning: Researchers can employ AI and machine learning algorithms to analyze extensive datasets from individuals exposed to asbestos. These algorithms can identify complex patterns and interactions between various factors, including exposure levels, duration, asbestos types, and genetic predisposition. These advanced analytical approaches should be used in synergy with traditional statistical methodologies and with Bayesian (causal) discovery to address the complexity of multidimensional datasets. The hope is that they can help predict the impact of asbestos more accurately and identify personalized thresholds. The promise in this area, however, has yet to be realized.
  ○ *Example Innovation:* Karunakaran et al. (49) used generative AI to make protein-protein interactions (PPI) and genomic interactions understandable to translational researchers with Wiki-Pi. The authors also discussed this approach for genomic data interpretation with ToxicoGenomica (see above) if the PPI pilot proves promising for public genomic and proteomic data. Other possibilities include deep learning using whole pathology slide images of mesothelioma cases and using causal discovery (advanced form of AI to query large cohorts) (50).
 • Multi-omics approaches: Data from genomics, proteomics, and metabolomics can be combined to create a comprehensive proﬁle of how asbestos exposure affects an individual's biology. This approach may reveal subtle molecular changes that precede mesothelioma, leading to the identiﬁcation of critical exposure thresholds.
  ○ *Example Innovation*: Resources include the Human Atlas of Malignant Mesothelioma and Human Mesothelioma Interactome (https://mesotissue.org/; https://mesotheliomaspatialatlas.streamlit.app/; https://hagrid.dbmi.pitt.edu/wiki-MPM/) (49, 51).
 • Geographic and environmental factors: Researchers can consider the geographic and environmental context of asbestos exposure. Different regions have varying levels of asbestos types and environmental factors that inﬂuence mesothelioma risks [e.g., (39)]. Integrating geographical data into the assessment may improve exposure threshold estimates.
  ○ *Example Innovation:* Gao et al. (52) used industry and occupational exposures of patients in the mid-Atlantic region collected by the NMVB.
 • Public health surveillance: A comprehensive public health surveillance system that tracks mesothelioma cases, including non-occupational cases, and links them to exposure histories can be implemented. This system can provide valuable data for setting exposure limits for various scenarios. One approach would be to develop a National Mesothelioma Patient Registry, which was the conclusion of a Workshop held in 2018 (53).
  ○ *Possible Innovation*: Multiple researchers have noted that the US is only country without a government-sponsored national mesothelioma registry (52, 54).

**Figure 1:**
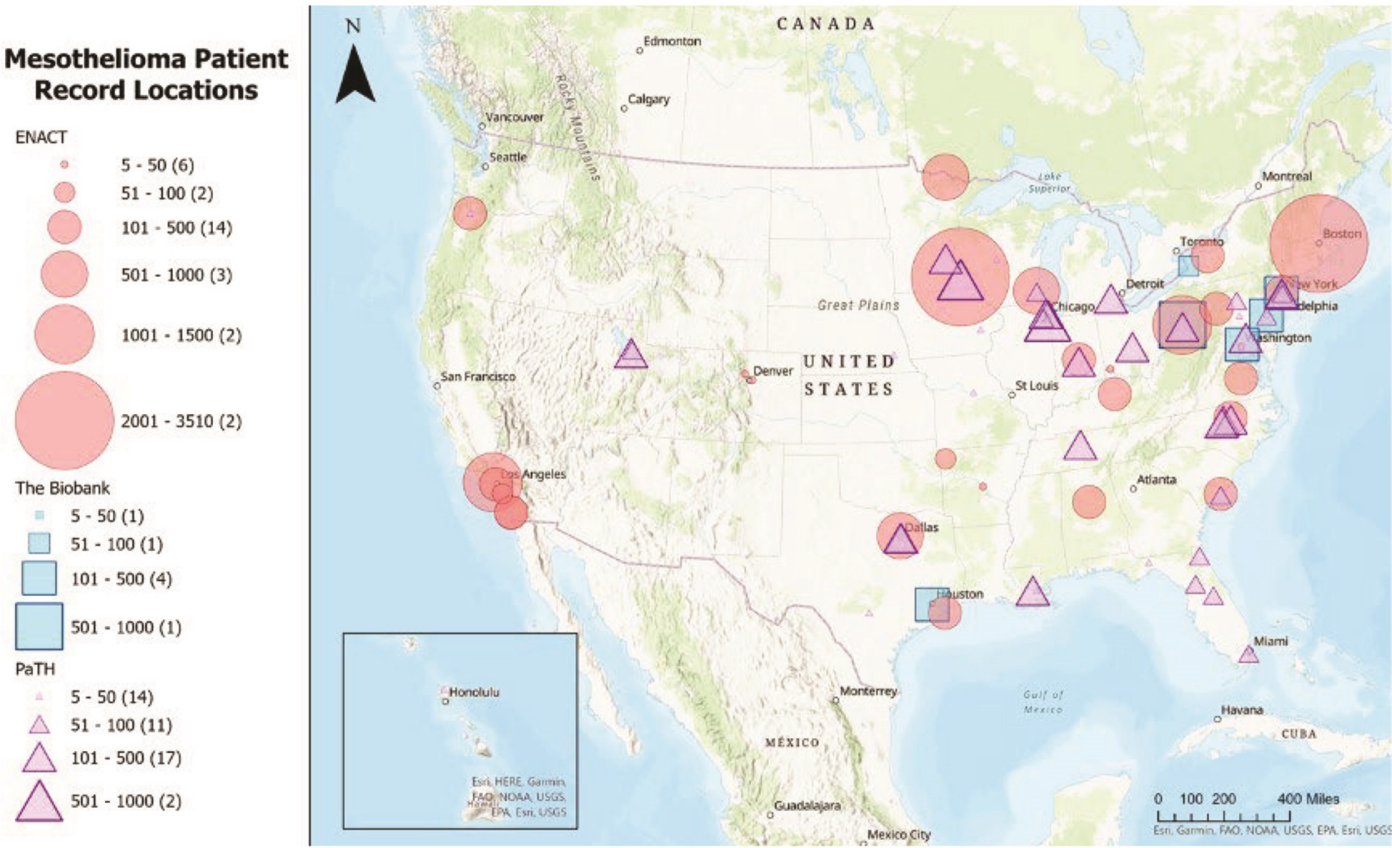
Mesothelioma patient record locations in the ENACT, NMVB, and PaTH/PCORnet data sharing networks. Source: This map was generated using data from Visweswaran et al. (55), Morrato et al. (56), Forrest et al. (57), and Amin et al. (58).

Determining precise thresholds for asbestos exposure leading to mesothelioma is a multi-faceted challenge, and it may require a combination of these novel approaches to improve our understanding and risk assessment capabilities. Collaboration between scientists, healthcare professionals, regulatory agencies, and the affected communities is essential to make progress in this ﬁeld. The NMVB provides a foundation for the formation of a National Mesothelioma Patient Registry and can leverage national Electronic Health Record (EHR) data sharing efforts (see Figure 1), including the Evolve to Next-Gen Accrual to Clinical Trials (ENACT) for Research (55, 56) and the Patient Centered Outcomes Research Network (PCORnet) (57, 58).

## Conclusions

One might ask whether there is any difference in protecting human health and safety with a threshold *vs*. non-threshold model. This concerns risk management actions that might be based on the risk assessment's findings, and so is beyond the scope of this paper. We note, however, that it is preferable to base risk management decisions on the soundest scientific footing available. If risk management practice assumes no threshold and infers risks at exposures below a true threshold, then actions might be counterproductive, perhaps incurring unnecessary costs and changing regulated processes such that they entail greater exposures to other hazards.

Thus, while there remain skeptics, the evidence indicates there are situations in which thresholds for asbestos and silica exist and methods to establish them quantitatively should be better developed. Researchers are urged to incorporate methodologies to include the possibility of a threshold so that data will exist to enable the setting of threshold-based standards where the data fit threshold models.

## Footnotes

^1^Goodman et al. Thresholds in epidemiology and risk assessment: mesothelioma thresholds from every angle. (submitted).
